# Epidemiological profile of COVID-19 in the French overseas department Mayotte, 2020 to 2021

**DOI:** 10.2807/1560-7917.ES.2022.27.34.2100953

**Published:** 2022-08-25

**Authors:** Marion Subiros, Charlotte Robert De Latour, Fanny Parenton, Ibtissame Soulaimana, Youssouf Hassani, Renaud Blondé, François Pousset, Yvonnick Boué, Camille Estagnasie, Gonzague Martin-Lecamp, Abdoulahy Diallo, Lucas Balloy, Mohamadou Niang, Christophe Caralp, Aurélie Cann, Abdourahim Chamouine, Alice Miquel, Geneviève Dennetière, Julie Durand, Maxime Jean, Sophie Olivier, Louis Collet, Nicole Tayeb, Patrice Combe

**Affiliations:** 1 Santé publique France, Mamoudzou, Mayotte, France; 2 Centre Hospitalier de Mayotte, Mamoudzou, Mayotte, France; 3 Agence de santé Mayotte, Mamoudzou, Mayotte, France

**Keywords:** COVID-19, Beta variant, epidemiology, severity, surveillance, Mayotte, multi-systemic inflammatory syndromes, SARS-CoV-2

## Abstract

**Background:**

During the COVID-19 pandemic, national and local measures were implemented on the island of Mayotte, a French overseas department in the Indian Ocean with critical socioeconomic and health indicators.

**Aim:**

We aimed to describe the COVID-19 outbreak in Mayotte from March 2020 to March 2021, with two waves from 9 March to 31 December 2020 and from 1 January to 14 March 2021, linked to Beta (20H/501Y.V2) variant.

**Methods:**

To understand and assess the dynamic and the severity of the COVID-19 outbreak in Mayotte, surveillance and investigation/contact tracing systems were set up including virological, epidemiological, hospitalisation and mortality indicators.

**Results:**

In total, 18,131 cases were laboratory confirmed, with PCR or RAT. During the first wave, incidence rate (IR) peaked in week 19 2020 (133/100,000). New hospitalisations peaked in week 20 (54 patients, including seven to ICU). Testing rate increased tenfold during the second wave. Between mid-December 2020 and mid-January 2021, IR doubled (851/100,000 in week 5 2021) and positivity rate tripled (28% in week 6 2021). SARS-CoV-2 Beta variant (Pangolin B.1.351) was detected in more than 80% of positive samples. Hospital admissions peaked in week 6 2021 with 225 patients, including 30 to ICU.

**Conclusion:**

This massive second wave could be linked to the high transmissibility of the Beta variant. The increase in the number of cases has naturally led to a higher number of severe cases and an overburdening of the hospital. This study shows the value of a real-time epidemiological surveillance for better understanding crisis situations.

## Introduction

In March 2020, when the severe acute respiratory syndrome coronavirus 2 (SARS-CoV-2) which causes coronavirus disease (COVID-19), had spread widely [1,2], the World Health Organization declared a public health emergency of international concern. In France, more than 6,000 cases had been reported by 15 March, including 285 intensive care unit (ICU) admissions and 161 deaths [3]. At that time, the island of Mayotte, a French overseas department in the Indian Ocean, was little affected. However, several socioeconomic and health indicators suggested a potential for SARS-CoV-2 to rapidly spread through the territory. Mayotte is one of the most densely populated territory in France (279,471 inhabitants) and the majority of the population (77%) lives below the poverty line [4,5]. Half of the houses are overcrowded, containing only one or two rooms, and a third of the houses have no access to running water. Immigration from Comoros has put additional strain on the demographic pressure [6]. The level of literacy is low [7], making awareness campaigns about the disease complex. Medical density is also low: 54 general practitioners per 100,000 inhabitants, and one single hospital, which has only 163 medical beds and 16 intensive care beds.

With half of the population under 18, elderly people (≥ 65 years) represent only 2.7% (ca 7,000) of the population [8]. Nevertheless, the prevalence of risk factors for severe forms of COVID-19 is high: two thirds of adults are overweight, 48% of 30–69-year olds are estimated to have high blood pressure and the prevalence of diabetes is 12% in adults (data not shown).

The COVID-19 outbreak started while Mayotte was facing an unprecedented dengue outbreak. The island was experiencing work interruption, overcrowded primary care services, hospitalisations, and deaths. It was the largest dengue outbreak described in Mayotte with more than 4,000 laboratory-confirmed cases, 440 hospitalisations and 21 deaths [9].

To understand COVID-19 in Mayotte, surveillance and investigation/contact tracing systems were set up by Santé publique France in collaboration with its partners. This article describes the epidemiology of COVID-19 in Mayotte during the first year of the pandemic from 9 March 2020 to 14 March 2021 (i.e., week 11 2020 to week 10 2021). Two waves are described here: one in 2020 (9 March 2020 to 31 December 2020) with SARS-CoV-2 wild type circulation and one in 2021 (1 January 2021 to 14 March 2021) when the Beta variant (Phylogenetic Assignment of Named Global Outbreak (Pango) lineage designation B.1.351) emerged [10].

## Methods

### Case definitions

COVID-19 surveillance was based on case definitions established by Santé publique France [11]. A probable case was defined as a person with clinical and chest computed tomography (CT) signs suggestive of COVID-19. A confirmed case was a person, symptomatic or not, with a laboratory-confirmed infection with SARS-CoV-2 by polymerase chain reaction (PCR) or rapid antigen test (RAT). A suspected case was a person with an acute respiratory infection of sudden onset and symptoms of fever and/or cough, chest pain, dyspnea, sore throat, nasal congestion, asthenia, headache, myalgia of any severity.

### Surveillance system

Several virological and epidemiological indicators were monitored during the surveillance of COVID-19 in order to investigate the outbreak’s dynamics (Table 1). These included the incidence rate (IR), testing rate (TR), and test positivity rate (PR). The distribution of cases by age group was estimated according to the regional population census [8]. A local database was used specifically for monitoring clusters. Hospitalisation in Mayotte Hospital Centre (CHM), and mortality indicators helped to assess the outbreak’s severity (Table 1).

**Table 1:** COVID-19 surveillance system in Mayotte, France, 2020–2021

Type of surveillance	Data source	Data used	Indicators for monitoring the epidemiological situation
Virological surveillance of SARS-CoV-2	Local laboratory databases from 9 March to 1 November 2020: - Mayotte Hospital Centre - Private laboratory in Mayotte - Réunion Hospital (tests for Réunion-Mayotte travellers between 21 July 2020 and 19 September 2020); SI-DEP from 2 November 2020 to 14 March 2021	Test results for SARS-CoV-2 and age of patients	COVID-19 testing rate per 100,000 inhabitants Number of new COVID-19 confirmed cases; COVID-19 incidence rate per 100,00 inhabitants (alert threshold of 50 per100,000); SARS-CoV-2 positivity rate (alert threshold of 10%)
	Viral whole genome sequencing or targeted mutation screening databases (2021)		SARS-CoV-2 strains proportions
Epidemiological surveillance of COVID-19 clusters	Clusters database	Type of clusters, number of cases per cluster, degrees of criticality (based on number of cases, hospitalisation rate, mortality and risk of dissemination)^a^	Characteristics of COVID-19 clusters
Surveillance of the activity of Mayotte Hospital Centre (CHM) emergency unit	OSCOUR	Admissions coded as ‘suspected COVID-19’ in the emergency unit^b^	Weekly number of admissions for COVID-19 suspicion in the emergency unit^b^
			Rate of hospitalisation following admission in the emergency unit^b^
Surveillance of COVID-19 cases, hospitalised at CHM, all units combined	SI-VIC^e^	Number of COVID-19 cases hospitalised in CHM^b^	Weekly number of COVID-19 cases hospitalised in CHM^b^
Epidemiological surveillance of COVID-19 severe cases, hospitalised in CHM ICU	Database for severe cases hospitalised in ICU	Number of COVID-19 cases hospitalised in ICU	Weekly number of COVID-19 cases hospitalised in ICU
		Sociodemographic, clinical and biological data	Characteristics of COVID-19 cases admitted in ICU
Mortality surveillance	Death certificates	Number of deaths among COVID-19 cases^c^	Mortality rate among COVID-19 cases hospitalised in CHM^c^
	Investigation and contact tracing database		
	SI-VIC		

CHM: Mayotte Hospital Centre; COVID-19: coronavirus disease; ICU: intensive care unit; OSCOUR: Organisation de la Surveillance Coordonnée des Urgences (national surveillance system for hospital emergency units); SARS-CoV-2: severe acute respiratory syndrome coronavirus 2; SI-DEP: système d’Informations de Dépistage (national database recording all test results in France); SI-VIC: Système d’Information d’identification unique des VICtimes (national database on conventional and intensive care hospitalisations).

^a^ Santé publique France guide for identification and investigation of COVID-19 clusters is available from [13].

^b^ Without any notion of link between infection and hospitalisation.

^c^ Without any notion of link between infection and death.

### Testing strategy

Mayotte has two laboratories: the CHM laboratory and a private laboratory. These laboratories started performing SARS-CoV-2 PCR testing in week 5 2020 and week 17 2020, respectively. The testing strategy was based on the epidemiological situation, human and logistical resources. Initially and during the first 5 months, laboratories could perform up to 200 tests per day. Any patient over 6 years of age meeting the COVID-19 suspected case definition was eligible for testing. Thus, only symptomatic patients were tested. Only health authorities could request contact testing during their investigations. From week 31 2020, with a doubling of testing capacities (up to 400 tests per day), household contact cases could be tested without delay, and within 5–7 days of the last contact for non-family contacts. From week 45 2020, access to tests was no longer restricted thanks to the wide deployment of RAT, for diagnosis, contact, travel, and investigations.

### Genomic surveillance

SARS-CoV-2 genomic surveillance was introduced in 2021 with the National Reference Centre for Respiratory Viruses in Paris. During 2 days in the first half of February, all usable SARS-CoV-2 positive samples were screened for variants of concern including the targeted mutations E484K, E484Q and L452R. Viral whole genome sequencing (WGS) was performed for ICU patients when the sample was usable. When WGS was not available, a targeted mutation screening was implemented.

### Investigations and contact tracing

An investigation platform was set up during the planning stage. A phone interview was conducted with each confirmed case to identify the origin of the contamination and explain preventive measures. We collected information on risk exposure in the 14 days before symptom onset. Contact tracing was performed to identify potential transmissions.

A cluster was defined by the occurrence of at least three confirmed or probable cases within a 7-day period, among individuals belonging to the same community or setting (school, workplace, prison etc.) or having participated in the same gathering of people, whether they know each other or not. For all clusters, authorities proposed that testing should be performed for all contacts.

Any confirmed symptomatic case was isolated for 7 days from the date of symptom onset. If fever persisted after 7 days, isolation was maintained until 48 h after fever stopped. Any confirmed asymptomatic case was isolated for 7 days from the date of the positive test. In case of symptoms, isolation was extended for a further 7 days from the date of symptom onset. For complex situations, home interventions were organised. This included people who did not understand the preventive measures, people who were stigmatised, and people who were unwilling to cooperate. The objective was to gain trust. Additional support could also be provided to families, such as food, water storage, hygiene equipment and emergency accommodation for isolation. The authorities made emergency shelter available for people in need, on a voluntary basis and during the contagious period.

### Statistical analysis

The study presents a descriptive analysis of the epidemiological indicators. Comparative analyses between the two COVID-19 waves were performed for TR, mean age of death, in-hospital mortality rate and characteristics of severe cases admitted to ICU with severe pulmonary COVID-19.

Statistical analysis was performed using RStudio software version 1.4 [12]. Categorical variables were compared using the chi-squared or Fisher's exact test. Student’s t-tests and Mann–Whitney tests were used to compare quantitative variables. The limit of significance was set at p < 0.05. Medians are presented with the first and third quartile.

## Results

### Outbreak dynamics, case and cluster characteristics

**Figure 1:**
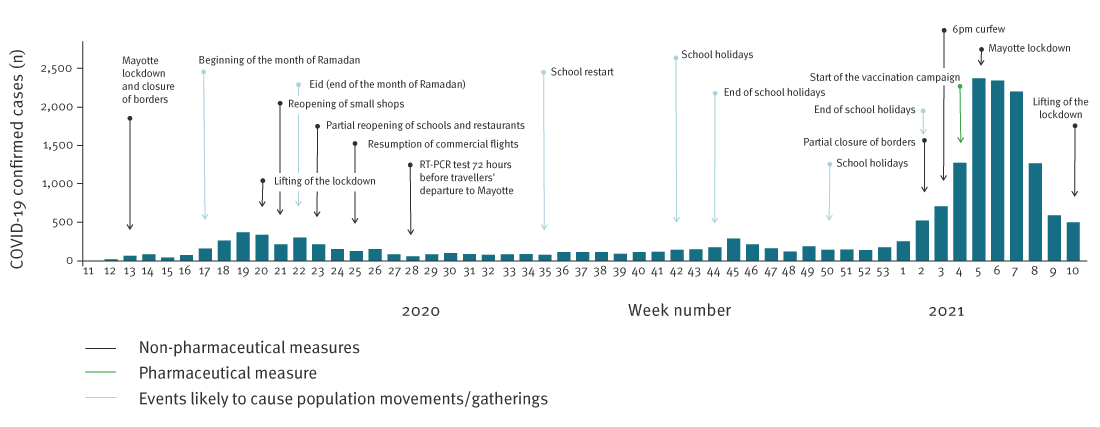
Epidemic curve of confirmed COVID-19 cases by sampling date, timeline of public health measures and significant local events, Mayotte, week 11 2020–week 10 2021 (n = 18,131)

During the study period, 18,131 COVID-19 cases were laboratory confirmed in Mayotte. The first case was diagnosed on 13 March 2020, 4 days before the national and overseas lockdown (Figure 1). It was an imported case from mainland France. Several measures were set up in Mayotte. The island followed the 2020 national lockdown and set up local measures such as closing of borders, testing all persons travelling to Mayotte when borders were reopened, curfew, a second local lockdown, and a vaccination campaign (Figure 1).

COVID-19: coronavirus disease.

**Figure 2:**
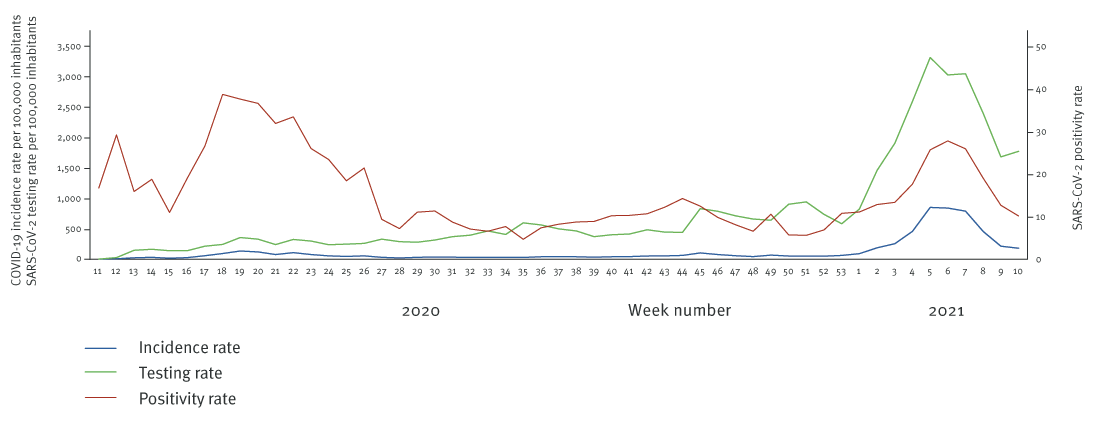
COVID-19 incidence and testing rate (per 100,000 inhabitants), SARS-CoV-2 positivity rate, Mayotte, week 11 2020–week 10 2021

COVID-19: coronavirus disease; SARS-CoV-2: severe acute respiratory syndrome coronavirus 2.

During the first wave, the virus spread rapidly and the first epidemic peak was reached in May 2020. The IR was 133 cases per 100,000 inhabitants in week 19 2020 (373 confirmed cases). The PR was particularly high, with a peak in week 18 2020 at 39%. The peak was first observed in people aged 45 years and over, then in 15–44-year olds. For children under 15 years old, the IR was never higher than 5 per 100,000 and the PR remained below 10%. From May 2020, IR and PR have progressively decreased to below the alert thresholds of 50 per 100,000 and 10%, respectively, by the beginning of July (week 27 2020) (Figure 2).

From July to December, viral spread continued at a low level with a mean IR of 46 per 100,000 and a mean PR of 9%. In early 2021, Mayotte experienced a rapid and intense deterioration of the situation. IR doubled and PR tripled between mid-December and mid-January. During this second wave, the epidemic peak was reached in week 5 2021 with an IR of 851 per 100,000 (2,378 new confirmed cases). This peak was particularly marked in the elderly population (≥ 65 years) (IR = 19,276/100,000). For the first time, the IR increased in children under 15 years old and peaked at 654 per 100,000 in week 5 2021. From the beginning of 2021, PR increased rapidly in all age groups, peaking at 28% in week 6 2021 (Figure 2). In February 2021, the Beta variant was detected in more than 80% of SARS-CoV-2 positive samples (150/172).

**Figure 3:**
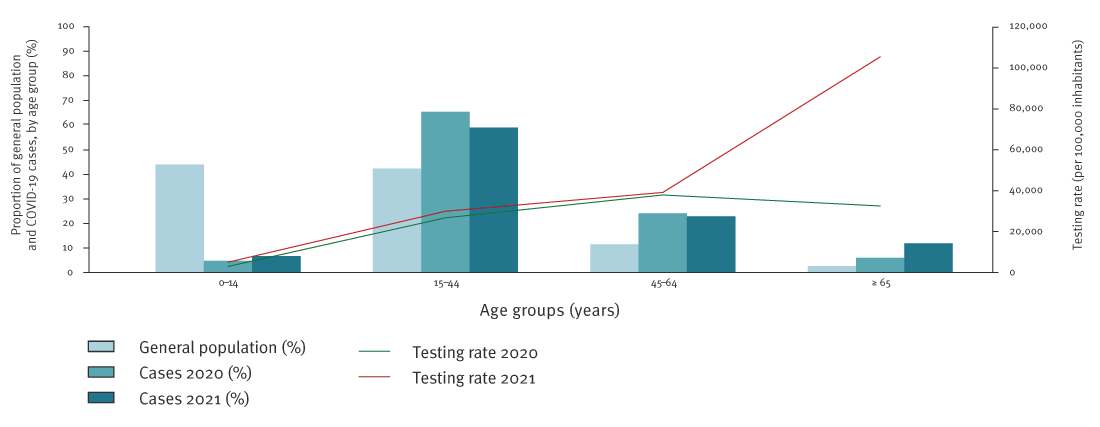
Distribution of general population, COVID-19 confirmed cases and SARS-CoV-2 testing rate (per 100,000 inhabitants), by wave and age group, Mayotte, week 11 2020–week 10 2021

COVID-19: coronavirus disease; SARS-CoV-2: severe acute respiratory syndrome coronavirus 2.

During the study period, 122,774 tests were performed, including 77,067 PCR and 45,707 RAT. The TR was low during the first weeks of the outbreak. It reached a peak in week 19 2020 with 355 tests per 100,000 inhabitants. Then, the TR increased almost constantly during one year, accelerating in week 45 2020 due to the deployment of RAT. At the end of 2020, the TR had increased tenfold from the start of the first wave. The TR peak was reached in week 5 2021 with 3,314 tests per 100,000 inhabitants (Figure 2). The TR increased significantly, both in children and in elderly people, in the second wave compared to the first. Between 2020 and 2021, the TR increased by a factor of 1.7 and 3.2, respectively, in these populations, while it remained stable among 15–64-year olds (Figure 3).

During the study period, 185 clusters were identified: 99 clusters in public and/or private companies, 31 in associations, 23 in family environments, 12 in health establishments, nine in schools and universities, four in connection with private and/or public events, three in the prison, two in a medico-social structure, one in a military regiment and one on a plane. In total, 1,884 cases were linked to these clusters. Most of the clusters had a ‘moderate’ risk degree (59 'high', 72 'moderate' and 55 'limited' risk) [13]. The average number of cases per cluster was 10. Of the 185 clusters, the prison clusters identified the most cases (307 cases, of which there were 236 cases in June 2020 and 71 in February 2021). The second largest cluster was in a military regiment (52 cases in October 2020) followed by one in a municipality in the north of the island (47 cases identified in August 2020).

### Severity of the epidemic

Over the study period, 2,292 visits for a ‘COVID-19 suspected case’ were recorded in the CHM emergency unit (1,759 in 2020 and 535 in 2021). A peak of 223 visits was reached in week 19 2020 with a 10% hospitalisation rate (n = 23). During the second wave, there was a peak in week 6 2021 of 134 visits with a 35% hospitalisation rate (n = 47).

In total, 1,524 persons with a COVID-19 infection (8.4% of all laboratory-confirmed cases) were hospitalised at the CHM during the study period (all causes, all lengths of stay, without any notion of link between infection and hospitalisation). There was a total of 613 hospitalisations in 2020 and 911 in 2021.

**Figure 4:**
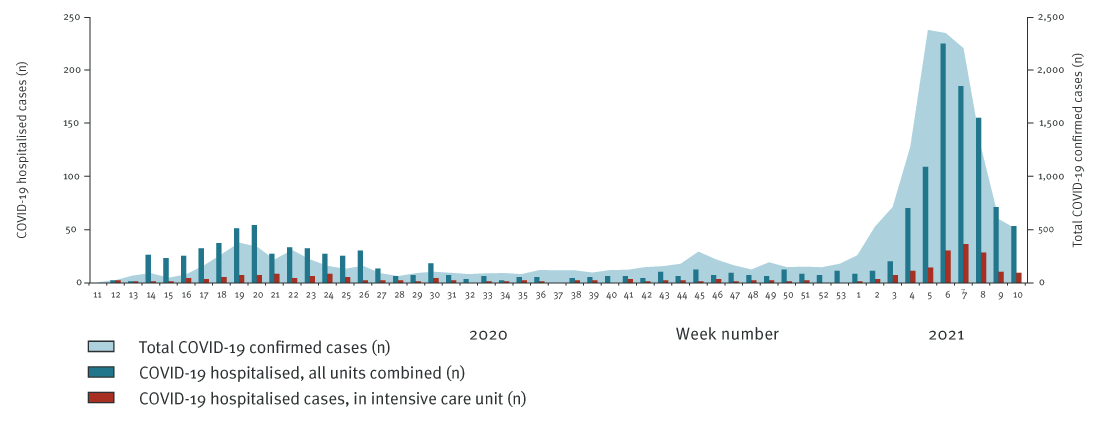
Weekly evolution of total COVID-19 cases and new COVID-19 cases hospitalised at Mayotte Hospital Centre, week 11 2020–week 10 2021 (n = 1,524)

During the first wave, a peak of weekly new admissions was observed in week 20 2020 (54 patients hospitalised, including seven in the ICU). The peak in ICU admissions was reached in week 21 2020, with eight new patients admitted (Figure 4). The number of new hospital admissions remained low during the austral winter. During the second wave in 2021, the hospital was rapidly overloaded with a peak of new admissions reached in week 6 2021. Here, 225 patients were hospitalised, including 30 in the ICU. The peak in ICU patients was reached in week 7 2021 with 36 new admissions.

COVID-19: coronavirus disease.

### Characteristics of cases hospitalised in the intensive care unit

Among the 18,131 laboratory-confirmed cases, 253 (1.4%) patients were admitted to the ICU (104 in 2020 and 149 in 2021). There were 195 cases of severe pulmonary illness, 44 cases of asymptomatic infection (other reason for admission to ICU) and 14 cases of multi-systemic inflammatory syndrome associated with SARS-CoV-2 infection (six adults and eight children). Ten pregnant women were admitted to ICU with COVID-19, two in 2020 and eight in 2021, all with a favourable outcome.

Most ICU patients (n = 248) had laboratory confirmation of infection. The five patients without laboratory confirmation had clinical and radiological findings strongly suggestive of SARS-CoV-2 infection. For 106 patients a chest CT scan was performed, suggestive of COVID-19 lesions for 80 of them (75%). During the second wave, viral WGS was performed for 23 patients in the ICU. The majority (n = 20) were infected with the Beta variant. Three ICU patients, all admitted in January 2021, had the wild strain (S614G) with European distribution. Four ICU patients underwent a targeted mutation screening, suggesting infection with the Beta variant.

Patients with severe pulmonary illness (n = 195) had a median age of 59 years (IQR:48–67) and almost half were under 60 years old (49.2%) (Table2). In the second wave, a significantly higher proportion of patients < 60 years was observed compared with the first wave (p = 0.04). The sex ratio M/F was 1.7. The median time from symptom onset to admission to ICU was 6 days (IQR: 4–9). It was significantly longer in the second wave (7 days (IQR: 4–9)) compared with the first (5 days (IQR: 3–7)).

**Table 2:** Characteristics of severe cases admitted to ICU with pulmonary COVID-19, Mayotte Hospital, first wave (9 March 2020–31 December 2020) vs second wave (1 January 2021–14 March 2021) (n = 195)

Characteristics	Number of cases for which information is available (N)	All cases (n = 195)		Cases first wave (n = 60)			Cases second wave (n = 135)				p value
		n	%	n	%		n			%	
Sex ratio male/female	195	1.7		2			1.6				NA
Female	NA	72	37.0	20		33.0	52		39.0		0.5
Male		123	63.0	40		67.0	83		61.0		
Median age (IQR) in years	195	59.2 (48.4–66. 6)		62.2 (51.4–68. 4)			57.5 (47.2–66.1)				0.05
0–9	NA	0	0.0	0		0.0	0		0.0		NA
10–19		1	0.5	0		0.0	1		0.7		
20–29		6	3.1	1		1.7	5		3.7		
30–39		9	4.6	2		3.3	7		5.2		
40–49		36	18.5	9		15.1	27		20.0		
50–59		49	25.2	13		21.6	36		26.7		
60–69		64	32.8	22		36.7	42		31.1		
70–79		27	13.8	11		18.3	16		11.9		
80–89		3	1.5	2		3.3	1		0.7		
0–59		96	49.2	23		38.3	73		54.1		0.04
≥ 60		99	50.8	37		61.7	62		45.9		
Comorbidity^a^	192	NA									
No comorbidity	NA	20	10.0	6		10.0	14		11.0		0.9
At least one comorbidity, including:		172	90.0	54		90.0	118		89.0		
Diabetes	NA	99	51.0	34		57.0	65		48.0		0.3
Hypertension		118	61.0	38		63.0	80		59.0		0.6
Obesity (BMI ≥30 kg/ m ^2^)	187	89	48.0	21		38.0	68		52.0		0.07
Morbid obesity (BMI ≥ 40 kg/m ^2^)	187	21	11.2	4		4.1	17		13.0		0.2
Cardiac pathology	NA	12	6.2	5		8.3	7		5.2		0.5
Pulmonary pathology		18	9.2	6		10.0	12		8.9		0.8
Renal pathology		25	13.0	10		17.0	15		11.0		0.3
Immunodeficiency		6	3.1	1		1.7	5		3.7		0.7
Cancer		10	5.1	3		5.0	7		5.2		> 0.9
Neuromuscular pathology		13	6.7	5		8.3	8		5.9		0.5
Liver disease		3	1.5	3		5.0	0		0.0		0.03
Other co-morbidity		6	3.1	1		1.7	5		3.7		0.7
Acute respiratory distress syndrome^b^	186	NA									
Severity: minor	NA	4	2.2	3		5.6	1		0.8		0.04
Severity: moderate		58	31.0	12		22.0	46		35.0		
Severity: severe		124	67.0	39		72.0	85		64.0		
Respiratory support (the most invasive during the stay)	187	NA									
O_2_ therapy /high flow O_2_ / non-invasive ventilation	NA	50	26.7	19		34.5	31		23.5		0.1
Invasive ventilation / extracorporeal membrane oxygenation		137	73.3	36		65.5	101		76.5		
Timeline											
Median time from symptom onset to ICU admission (IQR) in days	187	6 (4–9)		5 (3–7)			7 (4–9)				0.01
Median length of stay (IQR) in days	189	12 (5–23)		11 (4–23)			13.5 (8–22)				0.07
Median time from symptom onset to death (IQR) in days	60	19.5 (14–26)		15.5 (11–24)			20.5 (15–26)				0.1
Evolution	189	NA									
Discharge from intensive care unit	NA	126	66.7	39	66.1		87	66.9			0.9
Death		63	33.3	20	33.9		43	33.1			

BMI: body mass index; ICU: intensive care unit; IQR: interquartile range; NA: not applicable.

^a^ A patient may have several of the comorbidities listed in the table.

^b^ Severity level calculated according to Berlin criteria [14].

Among the 195 patients with severe pulmonary illness, 172 patients (90%) had at least one comorbidity, including high blood pressure (n = 118; 61%), diabetes (n = 99; 51%) or obesity (n = 89; 48%). The comorbidity profile of the patients was not different between the two waves. However, a comparison of median body mass index (BMI) showed a difference at the limit of statistical significance: 28 kg/m^2^ (IQR: 24–34) in 2020 vs 30 kg/m ^2^ (IQR: 26–35) in 2021 (p = 0.05).

Most of these patients (186/195) developed acute respiratory distress syndrome (ARDS), with two thirds (n = 124) developing a severe form according to the Berlin criteria [14]. Moderate forms of ARDS were more prevalent in the second wave. Invasive ventilation (intubation or extracorporeal assistance) was necessary for 137 (70%) of the patients with pulmonary illness admitted to ICU. For eight patients, no ARDS was reported. But they had pulmonary signs and were in a serious condition requiring intensive care (pregnant women, chronic obstructive pulmonary disease and cachexia).

The outcome was known for 97% of the patients (189/195). One hundred and twenty-six were discharged alive from the ICU, and 63 died in the unit. ICU mortality rate was 33%, with no significant difference between the two waves. The median length of stay in the ICU was 12 days (IQR: 5–23), with no significant difference between the two waves. For patients who died, the median time from symptom onset to death was 19 days (IQR: 14─25) (Table 2). A total of 76 patients were transferred to another ICU (medical evacuation by air to Réunion hospital) during the study period, almost all of them (n = 73) during the second wave.

### Mortality

During the study period, 145 deaths in people with a known COVID-19 infection were recorded. The male to female ratio of the patients who died was 1.5. There were 108 deaths at CHM, 13 deaths at home, 22 deaths in other hospitals following medical evacuation and two deaths in a public space. A total of 90 patients died in 2021, compared with 55 in 2020. There was no significant difference in age between those who died in the second wave (mean age 64 years) and those who died in the first wave (mean age 68 years) (p > 0.05).

The in-hospital mortality rate calculated for 2021 was 9.3% (85 deaths of 911 hospitalisations) and not significantly different to 2020 (7.4%, 45 deaths of 610 hospitalisations) (p = 0.2).

## Discussion

Here we describe a year-long COVID-19 epidemic in a vulnerable population in the Comoros archipelago. This work reflects independent scientific assessment designed to guide health policy. In Mayotte, authorities had to consider several obstacles. The first obstacle was socioeconomic fragility. At least 60% of dwellings had no running water, toilet or shower [4]. Some people could not isolate because there was not enough space in the house. Others had to leave the house to find daily subsistence resources despite the COVID-19 pandemic. In Mayotte, informal business accounts for two thirds of all occupations [15,16]. As in many countries, there were considerable implications of the pandemic for the informal economy [17]. This prompted the authorities to work on providing support (free water, food and face masks). The second obstacle was overcoming difficulties in institutional communication, such as misunderstanding the disease, the mechanism of clustering, contagiousness or asymptomatic infections and rumours contributing to the stigmatisation of people with COVID-19. Due to these obstacles, many clusters may not have been detected or were underestimated. These elements reaffirm the imperative need to develop trusted awareness-raising networks led by community and/or religious leaders in a community such as Mayotte where more than 95% of the people are Muslim [18]. They also suggest that the implementation of a lockdown, although effective in mainland France [19,20] may have been inadequate in Mayotte because of the difference in the population profile between mainland France and Mayotte.

Overall, it is likely that the scale of the COVID-19 epidemic in Mayotte was underestimated for many reasons. Seeking care was severely affected by lockdown restrictions and police controls, especially for illegal immigrants. In addition, many cases may not have been tracked and recorded into the database because they were never tested (epidemiological or clinical confirmation only). Finally, the testing strategy was not optimal as indications depended on local capacity. During the first months of the outbreak with limited test stocks, only symptomatic individuals were eligible for PCR. This has largely influenced the PR, which reached a peak of 39% in week 18 2020. In early 2020, the population was more concerned about the ongoing dengue outbreak than about COVID-19 and therefore did not accept the necessity of SARS-CoV-2 testing. This situation led to diagnostic difficulties [21].

If the magnitude of the second wave was much higher than the first one, it is difficult to conclude that the outbreak was of greater intensity on the basis of incidence data. Indeed, testing capacity was limited in the first wave and TR increased tenfold in the second wave. For example, between 2020 and 2021, while the TR of the elderly population had tripled, its IR had quadrupled. Nevertheless, viral spread had no major health impact until the end of 2020 when South Africa reported the emergence of the Beta variant in the Indian Ocean [10,22,23]. This variant was identified in Mayotte using screening and sequencing techniques at the national reference laboratory in Paris and showed dominance from February 2021. In the future, availability of these techniques on the island would allow for better preparedness.

Severity outcomes were the most reliable because they were based on the activity of the sole hospital of the island. However, hospitalisation and mortality rates do not take into account if hospitalisation was because of SARS-CoV-2 infection and future monitoring should provide this information. However, these indicators demonstrate a clear difference between the two waves, with a higher number of hospitalisations in 2021, especially in the ICU (149 admissions in 10 weeks in 2021 compared with 104 admissions in 43 weeks in 2020). In the second wave, the median age of patients hospitalised in the ICU decreased. There may be two explanations for this. Firstly, the number of infections among 15–44-year olds increased (IR doubled). Secondly, priority and massive vaccination from the end of January 2021 gave protection against a severe form of the virus for elderly persons. In both waves, 90% of patients admitted to ICU presented profiles likely to develop a severe illness [24]. Most of them developed a severe form of ARDS, although moderate forms were more prevalent in 2021. It is important to note that medical care was different in the second wave with better disease knowledge and an adaptation of the healthcare system. For example, the systematic introduction of corticosteroid therapy may have had an influence on the severity of ARDS, which was less likely to develop into a severe form [25]. In addition, the time between symptom onset and admission to ICU was longer in the second wave. This is a sign of healthcare system saturation, which is known to increase morbidity and mortality. That is why in 2021, the organisation of care was based on a new COVID ward in a surgery ward, cooperation with Réunion Island (for medical evacuations of serious cases) and a deployment of new tools (ambulance guard, home hospitalisation). For each wave, a systematic two-week time lag was observed between the pandemic peak in the general population, and the ICU admission peak. This is an important element in planning healthcare needs.

The limited severity of the first wave can be explained by the lower transmissibility of the wild strain of SARS-CoV-2 compared with the Beta variant, the introduction of severe restrictive public health measures as soon as the first case was discovered and the young age of the population. However, a number of COVID-19 multi-systemic inflammatory syndrome cases in children should be noted. Eight children required intensive care. Monitoring in the paediatric CHM unit revealed a high incidence of these forms, all with favourable outcomes [26,27].

Finally, our results are probably affected by the higher transmissibility of the Beta variant. This was also the conclusion of a study in South Africa, which showed that the Beta variant was associated with a 31% increased risk of in-hospital mortality [28,29].

## Conclusion

The information provided in this article is valuable for preparedness and future public health response in the region or for similar fragile populations in Europe. It shows how real-time epidemiological surveillance can be used in public health crisis situations to propose specific measures adapted to a socially vulnerable population. It demonstrates the urgent need to strengthen capacities to investigate cases and clusters during outbreaks also in resource-poor settings. This requires recruitment and training of actors in the field, e.g. identification and organisation of a network of community workers and the provision of advanced diagnostic techniques.
